# Theoretical derivation and experimental validation of tree detection rate in TLS-based forest plot surveys

**DOI:** 10.3389/fpls.2026.1869500

**Published:** 2026-08-17

**Authors:** Jingdong Sun, Yan Zhang, Minghui Zhao, Jiayue Xie, Yuhang Gao, Pei Wang

**Affiliations:** School of Science, Beijing Forestry University, Beijing, China

**Keywords:** forest inventory, mathematical model, plot characteristics, point cloud simulation, terrestrial laser scanning

## Abstract

Terrestrial laser scanning (TLS) has been widely used in forest inventories, where tree detection rate serves as a key indicator of scanning efficiency. However, limited research has focused on the quantitative correlation between plot design and detection rate. In this study, we systemically investigate how plot characteristics—including plot size, stem density, and diameter at breast height (DBH)—affect tree detection rate, using a combined approach of theoretical derivation, simulated point clouds, and field measurements. Based on scanning geometry, we derive an analytical detection rate formula that accounts for inter-tree occlusion and spatial tree distribution to estimate tree detection probability. For simulation analysis, we generate point clouds for 1600 virtual plots with controlled gradients of plot radius (10–25 m), stem density (200–1400 stems ha^-^¹), and DBH (0.1–0.4 m). The derived model is further validated using field TLS data from real forest plots. Results show that the theoretical detection rate estimates agree well with both simulated and field-measured values. The proposed formula can provide a reliable baseline for predicting detection rates in TLS plot scanning and support the optimization of forest inventory experimental design, demonstrating applicable practical value for operational forest surveys.

## 1 Introduction

Terrestrial laser scanning (TLS) has transformed forest inventory science over the past two decades, providing high-precision, non-destructive 3D characterization of forest structure at the individual tree level. This technology has become a standard tool for estimating critical forest biophysical parameters, including diameter at breast height (DBH) (Liu et al., 2018; Bruggisser et al., 2020), tree height (Pearse et al., 2019; Wang et al., 2019), leaf-filtering (Ali et al., 2024), and above-ground biomass (Fan et al., 2020; Cao et al., 2022; Ali et al., 2025). However, inter-tree occlusion represents an inherent physical limitation of TLS technology that no algorithmic advancement can fully overcome. Laser pulses are blocked by woody vegetation, leading to partial or complete occlusion of trees located behind foreground stems. This fundamental constraint typically requires multi-station scanning and computationally intensive point cloud registration, which can increase fieldwork time and substantially elevate operational costs. Consequently, maximizing tree detection rate in single-scan TLS surveys has emerged as a critical research priority for enhancing the cost-effectiveness and scalability of TLS-based forest inventories.

Tree detection rate, defined as the proportion of all trees in a sample plot that are successfully identified in TLS point clouds, serves as a key metric for assessing TLS survey performance. Alarmingly, published detection rates exhibit substantial variability, ranging from as low as 22% to perfect 100% across studies conducted over the past decades (**Table 1**). This substantial variability arises primarily from differences in data acquisition conditions and forest stand characteristics, including TLS instrument technology (phase-based vs. time-of-flight), scanning resolution, plot geometry (circular (Li et al., 2012; Liang and Hyyppä, 2013; Olofsson et al., 2014) vs. square (Dai et al., 2018)), plot dimensions, tree spatial distribution patterns, stem density, and terrain complexity. Despite this well-documented variability, the vast majority of existing research has focused disproportionately on developing more sophisticated tree detection algorithms, while few research efforts have been devoted to exploring the systematic effects of plot design and stand characteristics on detection performance. This critical knowledge gap has two profound implications: first, it renders cross-study comparisons of algorithm performance invalid, as differences in detection rates may reflect variations in experimental conditions rather than algorithmic superiority; second, it fails to provide forest practitioners with detailed, evidence-based guidelines for plot design optimization, thereby leading to suboptimal data collection, inefficient resource utilization, and inconsistent forest inventory results.

**Table 1:** Summary of plot characteristics and tree detection rates across studies.

Studies	Plot			Result
	Number	Size	Density (stem/ha)	Detection rate (%)
(Kankare et al., 2016)	1	30m × 30m	555.6	22
(Wilkes et al., 2017)	1	50m radius	130	40.2
(Seidel and Ammer, 2014)	5	15m radius	212-410	86.7-100
(Astrup et al., 2014)	1	30m radius	753	62.9–72.3
(Olofsson and Olsson, 2018)	18	30m × 33m or 25m×40m	207-570	59
(Kuronen et al., 2019)	2	0.5ha, 0.1ha	255, 950	100
(Thies and Spiecker, 2004)	2	20/50m radius	124/477	54/68
(Strahler et al., 2008)	6	0.13-0.2(ha)	1017-3281	42
(Maas et al., 2008)	20	12.62m radius	190000	94
(Liang and Hyyppä, 2013)	5	10m radius	605 ~ 1,210	95.3;73.4
(Brolly and Király, 2009)	2	0.5ha/0.5ha	1032/572	94;96
(Murphy et al., 2010)	1	0.5ha	1032	98
(Olofsson et al., 2014)	16	20m radius	358~1042	73.3
(Antonarakis, 2011)	1	31m × 16m	544	100
(Lovell et al., 2011)	3	30m×20m/25m×25m	18/133/56	99
(Yao et al., 2011)	4	10m × 10m	2000	92.75
(Li et al., 2012)	2	1ha/0.25ha	425/40	96/70
(Pueschel et al., 2013)	27	30m×30m ~ 30m×40m	705-835	98.8
(Pueschel, 2013)	6	25m × 25m	160-450	98
(Wang et al., 2016)	12	20m×20m/40m×40m	1154-2259	87-100

A handful of pioneering studies have begun to acknowledge the critical influence of plot and stand characteristics. Several studies have further focused on the quantitative assessment and correction of inter-tree occlusion effects. Adopting the tree count correction framework from Jupp et al. (2005), Lovell et al. (2011) quantified the single-scan occlusion effects in TLS and developed a correction method to enable the corrected TLS estimates to closely agree with conventional field measurements. Vauhkonen et al. (2012) demonstrated that plot structure significantly affects the accuracy of all commonly used single-tree detection algorithms through a comparative analysis across diverse forest types. Kankare et al. (2016) investigated how TLS point cloud sampling density influences tree detection rates and DBH measurement precision. Wilkes et al. (2017) provided a comprehensive review of TLS data collection protocols for forest inventories and their implications for above-ground biomass estimation. Seidel et al (Seidel and Ammer, 2014). quantified the shadowing effect on automated basal area measurements, which has long been unquantified in TLS forest applications, and proposed a novel correction method for laser unsampled areas caused by shadowing. Astrup et al. (2014) validated three model-based correction strategies to mitigate TLS nondetection bias, which employ detection probabilities derived from point transect sampling estimators, with calculations based solely on detected trees. Olofsson and Olsson (2018) conducted an innovative simulation study to clarify the nonlinear effects of plot size and stand density on stem visibility in single-scan TLS measurements, and proposed customized detection criteria and visibility-based tree weighting strategies to reduce occlusion-induced biases. On the basis of their findings, Kuronen et al. (2019) focuses on further improving the simple yet practical visibility-based correction method and develops a generalized estimator to retrieve forest attributes from single-scan TLS-derived stem coordinates and diameter data.

Despite the above achievements in TLS occlusion correction and forest attribute estimation, a generalizable theoretical framework for predicting tree detection rates based on fundamental scanning geometry and forest structural parameters remains lacking. Specifically, comprehensive quantitative associations between tree detection performance and key forest and plot factors—including plot size, individual tree diameter, and stand stem density—have not been fully clarified and validated in a holistic manner, leaving a critical research gap for the systematic optimization and practical application of single-scan TLS forest inventory techniques.

To address this critical knowledge gap, we present a comprehensive, multi-method investigation of how three fundamental plot and stand characteristics—plot radius, stem density, and mean DBH—quantitatively influence single-scan TLS tree detection rates. Our integrated research design combines three complementary approaches: (1) analytical theoretical modeling based on first-principles scanning geometry, which provides a concise, generalizable framework for predicting detection rates without the need for extensive empirical data; (2) controlled point cloud simulation experiments, which allow precise manipulation of individual variables while eliminating all confounding environmental factors, enabling the generation of large, statistically robust datasets spanning ecologically relevant parameter ranges; and (3) independent field validation using TLS data collected from natural forest plots at Yeyahu Wetland Park in Beijing, which confirms the real-world applicability of our theoretical findings and simulation.

This study makes three significant contributions to the field of TLS-based forest inventory. First, it develops the first analytical model derived from first principles that can accurately predict detection rates based solely on plot and stand characteristics. Second, it provides the first systematic, quantitative assessment of the independent effects of plot radius, stem density, and mean DBH on single-scan TLS detection rates across a wide range of ecologically relevant conditions. And third, it validates both simulation and theoretical results using independent field data. Our findings will enable standardized, objective comparisons of tree detection algorithms across different studies and provide forest researchers and practitioners with evidence-based guidelines for optimizing plot design and data acquisition protocols. Ultimately, this work will contribute to improving the cost-effectiveness, scalability, and reliability of TLS-based forest inventories globally, supporting more accurate assessments of forest carbon stocks and ecosystem services.

## 2 Experimental data

### 2.1 Simulating virtual sample plots

Inspired by the simulation framework of previous studies (Olofsson and Olsson, 2018), this study also constructs multi-gradient simulation scenarios with varying plot radii, tree DBH levels, and stand stem densities. Nevertheless, the present research differs distinctly in tree distribution patterns and parameter setting schemes. All simulations assumed a uniform spatial distribution of trees, with positions generated stochastically within the boundaries of each plot. Within each individual simulation run, all input parameters (plot radius, stem density, and DBH) were held constant to isolate their individual effects. Statistical inference regarding the impacts of each parameter and their interactions was derived from the full ensemble of simulated scenarios.

We conducted a series of idealized sample plot simulations using systematically varied combinations of plot geometry and tree trunk attributes, with detailed experimental specifications provided in **Table 2**. The first group employed a full factorial design with three discrete variables: circular plot radii of 10, 15, 20, and 25 m; stem densities of 200, 600, 1000, and 1400 stems ha^-^¹; and DBH values of 0.1, 0.2, 0.3, and 0.4 m. The second group retained identical plot radius and stem density levels as the first group, but assigned DBH values randomly within the 0.1–0.4 m range. Twenty replicate virtual plots were generated for each unique parameter combination to quantify simulation uncertainty.

**Table 2:** List of parameters and their purposes for simulated point cloud data.

Group	Plot radius (m)	Stem density (ha-1)	DBH values (m)	Combinations	Plots
1	10, 15, 20, 25	200, 600, 1000, 1400	0. 1, 0.2, 0.3, 0.4	64	1280
2	10, 15, 20, 25	200, 600, 1000, 1400	0.1∼0.4	16	320
Total				80	1600

For each sample plot, the theoretical number of trees, *N_cal_*, was calculated from plot radius R and stem density Dstem. Since this theoretical value is not necessarily an integer, we applied ceiling rounding to obtain an integer count. The actual number of trees generated in each simulated plot, *N_sim_*, was then set to this rounded value as follows (Equations 1–6).

(1) $$N_{cal}=\frac{\pi R^{2}D_{stem}}{10000}$$

(2) $$N_{sim}=ceil\left[N_{cal}\right]$$

The specific values of *N_cal_* and *N_sim_* for each combination of parameters are listed in **Table 3**. In total, 136,400 simulated tree trunk slices were generated across both Group 1 and Group 2.

**Table 3:** List of parameters and their purposes for simulated point cloud data.

Plot radius (m)	Stem density (ha-1)	N_cal_	N_sim_
10	200	6.28	7
	600	18.84	19
	1000	31.4	32
	1400	43.96	44
15	200	14.13	15
	600	42.39	43
	1000	70.65	71
	1400	98.91	99
20	200	25.12	26
	600	75.36	76
	1000	125.6	126
	1400	175.84	176
25	200	39.25	40
	600	117.75	118
	1000	196.25	197
	1400	274.75	275

### 2.2 Point cloud acquisition of real sample plots

Point cloud data acquisition for field validation plots was conducted from 8 to 10 August 2025 at Yeyahu Wetland Park, Yanqing District, Beijing by using Riegl-VZ400, whose characteristics are listed in **Table 4**. Five scanning stations were established per plot, each covering an area of approximately 50 m × 50 m and generating ~3.5 million points. Moderate overlap (30–40%) was maintained between adjacent stations to provide sufficient geometric constraints for subsequent registration, although all scans exhibited typical occlusion effects due to dense vegetation.

**Table 4:** Characteristics of RIEGL VZ-400 scanner.

Technical parameters	
Maximum scanning distance	600 m (Natural object reflectivity > 90%)
Vertical scan angle range	Total 100° (+60°/-40°)
Horizontal scan angle range	Max 360°
Range accuracy	5mm
Scan speed	3 lines/s to 120 lines/s (Vertical) 0°/s to 60°/s (Horizontal)
Angular resolution	Better than 0.0005°

**Figure 1:**
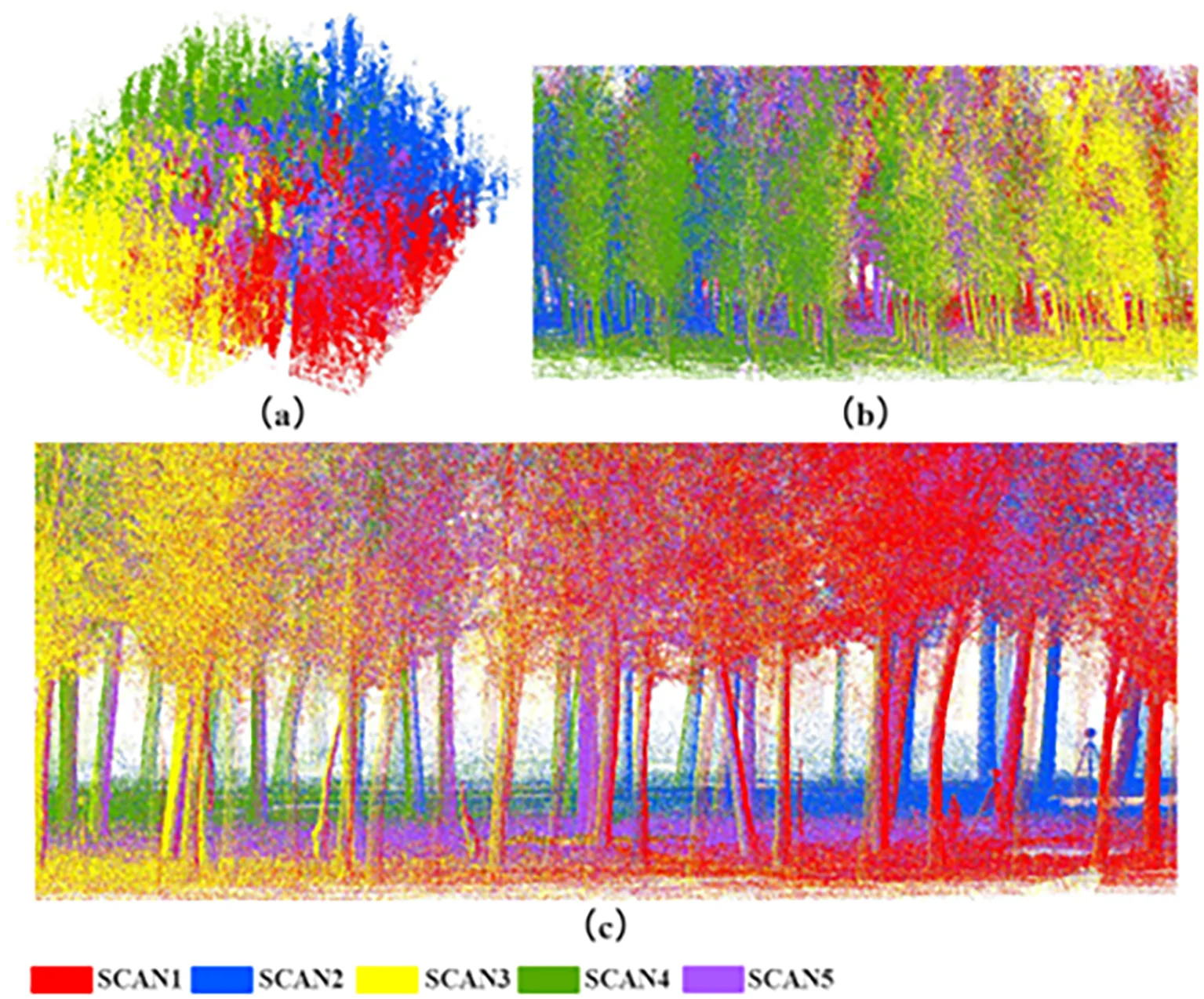
**(a)** Registered multi-scan point cloud; **(b, c)** side view multi-scan point cloud.

Multi-station point clouds were successfully registered, and the fused five-station dataset reconstructed a continuous and complete forest structural scene with naturally connected trunk morphologies and no detectable misalignment in overlapping regions. The registration quality fully satisfied the accuracy requirements for subsequent forest scene analysis (**Figure 1**). **Figure 1a** IS the top view of the plot, where red represents scan 1, blue represents scan 2, yellow represents scan 3, green represents scan 4, and purple represents scan 5. **Figures 1b, c** are side views of the plot from two different perspectives.

## 3 Methods

### 3.1 Overall scheme design

**Figure 2:**
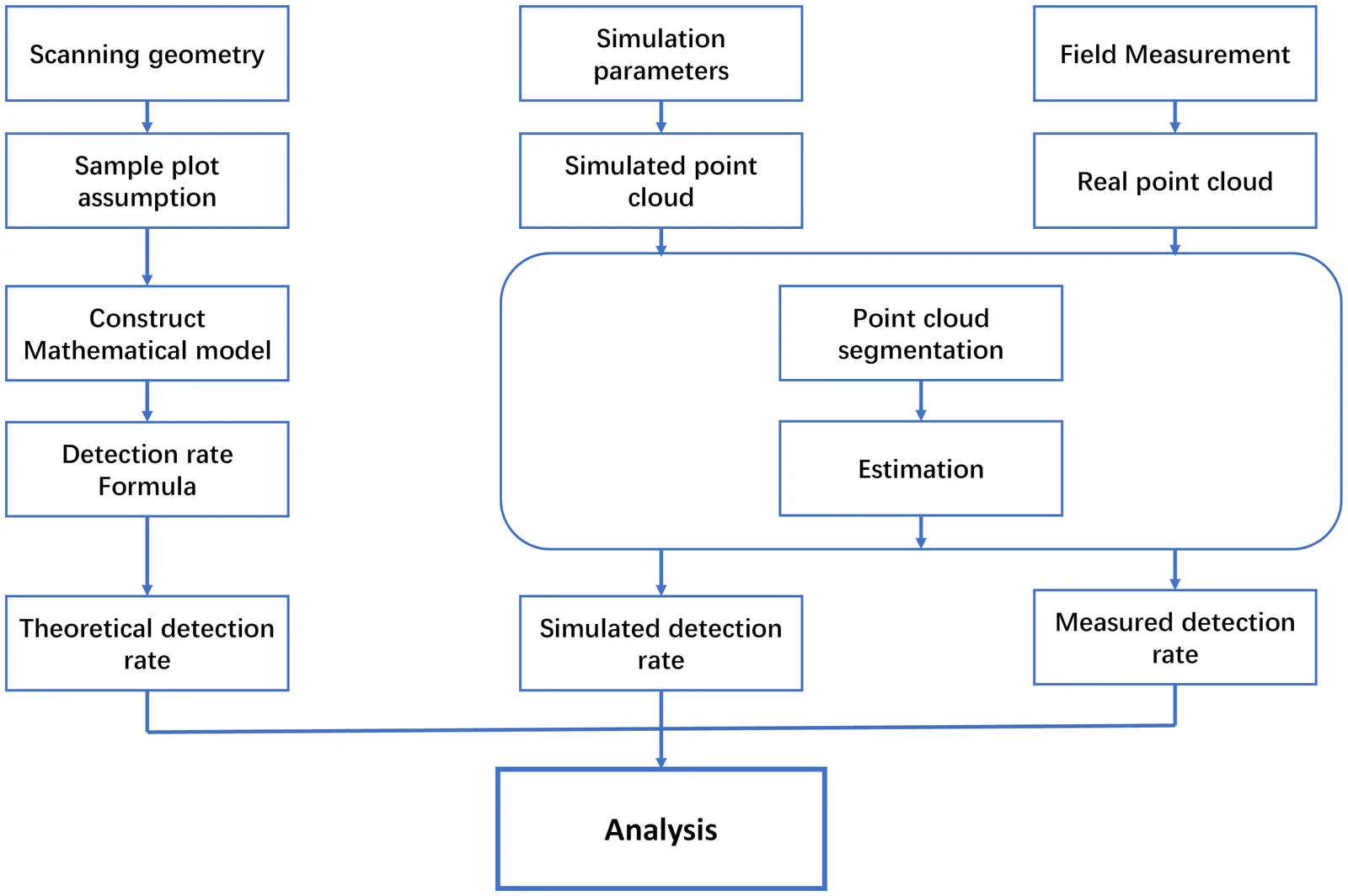
Flow chart of the research.

We adopted a three-tiered validation framework combining theoretical derivation, simulation-based assessment, and field validation to comprehensively evaluate tree detection rates. First, mathematical modeling was used to derive an analytical formula for the theoretical detection rate under defined plot conditions. Second, we assessed detection performance across the full range of simulated forest structural scenarios. Third, field validation was performed using TLS data from Yeyahu Wetland Park to confirm the generalizability of our findings. The complete research workflow and key results are summarized in **Figure 2**.

### 3.2 Theoretical derivation

In this section, we derive an analytical estimate of tree detection rate under occlusion conditions based on a set of simplifying assumptions. To construct a tractable mathematical model, we assume that:

1. The sample plot is a perfect circle of radius R;
2. All trees have an identical DBH (= 2*r*_0_);
3. There are *N_sim_* trees within the plot boundary;
4. Trees are randomly and uniformly distributed across the plot area.

**Figure 3:**
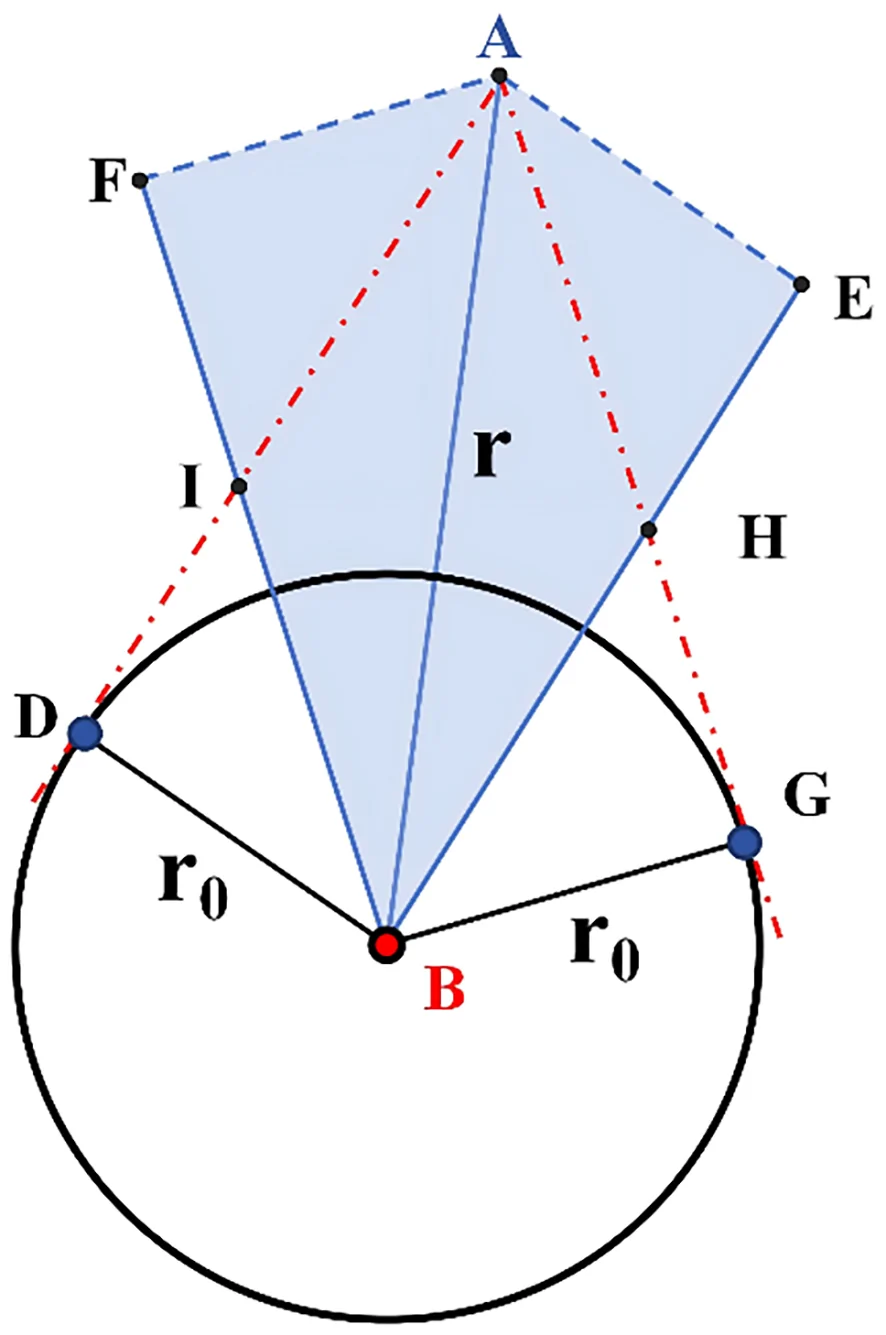
The variation in detection rate under bivariate conditions.

To illustrate the geometric principles underlying occlusion-induced detection failure, we present a schematic diagram of the LiDAR scanning geometry in **Figure 3**. The key elements and geometric relationships are defined as follows:

A: LiDAR scanner position;

B: Center of a tree trunk;

r: Distance between the scanner (A) and the tree center (B);

*r*_0_: Radius of the tree trunk (distance between B and C);

Meanwhile, the following geometric constructions are used in the derivation: Lines AD and AG are tangents to the circle centered at B (representing the tree trunk), with D and G being the points of tangency. Lines BE and BF are parallel to AD and AG, respectively. Lines AE and AF are parallel to BD and BG, respectively.

Based on the principle of estimating probability via frequency, the detection rate (*DR*) can be estimated by taking the mathematical expectation of the detection probability of an individual tree using TLS LiDAR. We can estimate *DR* by

(3) $$DR=\int_{1}^{R}T\left(r\right)P\left(r\right)$$

where T(r) is the probability of tree presence within an annular micro-element with radius ranging from r to r+dr centered on the scanner, and P(r) is the probability that the tree is not obscured by other trees.

First, we have

(4) $$T\left(r\right)=\frac{2r}{R^{2}}dr$$

Let *S_occ_* denote the occlusion domain, i.e., the region where any other tree will block the line of sight to the target tree. Therefore, the probability that the target tree is occluded, denoted *P_occ_*, is equal to the ratio between the area of *S_occ_* and the total sample plot area *S*.

Geometrically, *S_occ_* approximately corresponds to the area of the quadrilateral AFBE in **Figure 3**, with the only difference arising from the overlapping region between the quadrilateral and the circular boundary. In practice, since the radius of the circle (representing the tree) is much smaller than its distance to the LiDAR sensor, the overlapping area is negligible. Therefore, the area of the quadrilateral AFBE can be used as an accurate approximation of *S_occ_*.

(5) $$P_{occ}=\frac{S_{occ}}{S}=\frac{r_{0}\sqrt{r^{2}-r_{0}^{2}}}{\pi R^{2}}$$

Then, the probability that the tree is not obscured by other trees, P(r)

(6) $$P\left(r\right)={\left(1-Pocc\right)}^{N_{sim}-1}$$

Then we have

(7) $$DR=\int_{1}^{R}\frac{2r}{R^{2}}{\left(\frac{\pi R^{2}-r_{0}\sqrt{r^{2}-r_{0}^{2}}}{\pi R^{2}}\right)}^{N_{sim}-1}dr$$

Therefore, for TLS surveys of forest plots, given a known plot radius R, stem density, and tree DBH, we can use the above equation to predict the theoretical tree detection rate for that plot. The lower integration limit is defined based on two practical considerations. First, most laser scanners have an inherent near-field blind zone in practical operation. Second, scanners are typically not deployed in immediate proximity to surrounding trees during field setup. While the lower integration limit is set to 1 meter in the equation above, this threshold can be flexibly adjusted to accommodate specific real-world conditions.

### 3.3 Sample plot simulation

**Figure 4:**
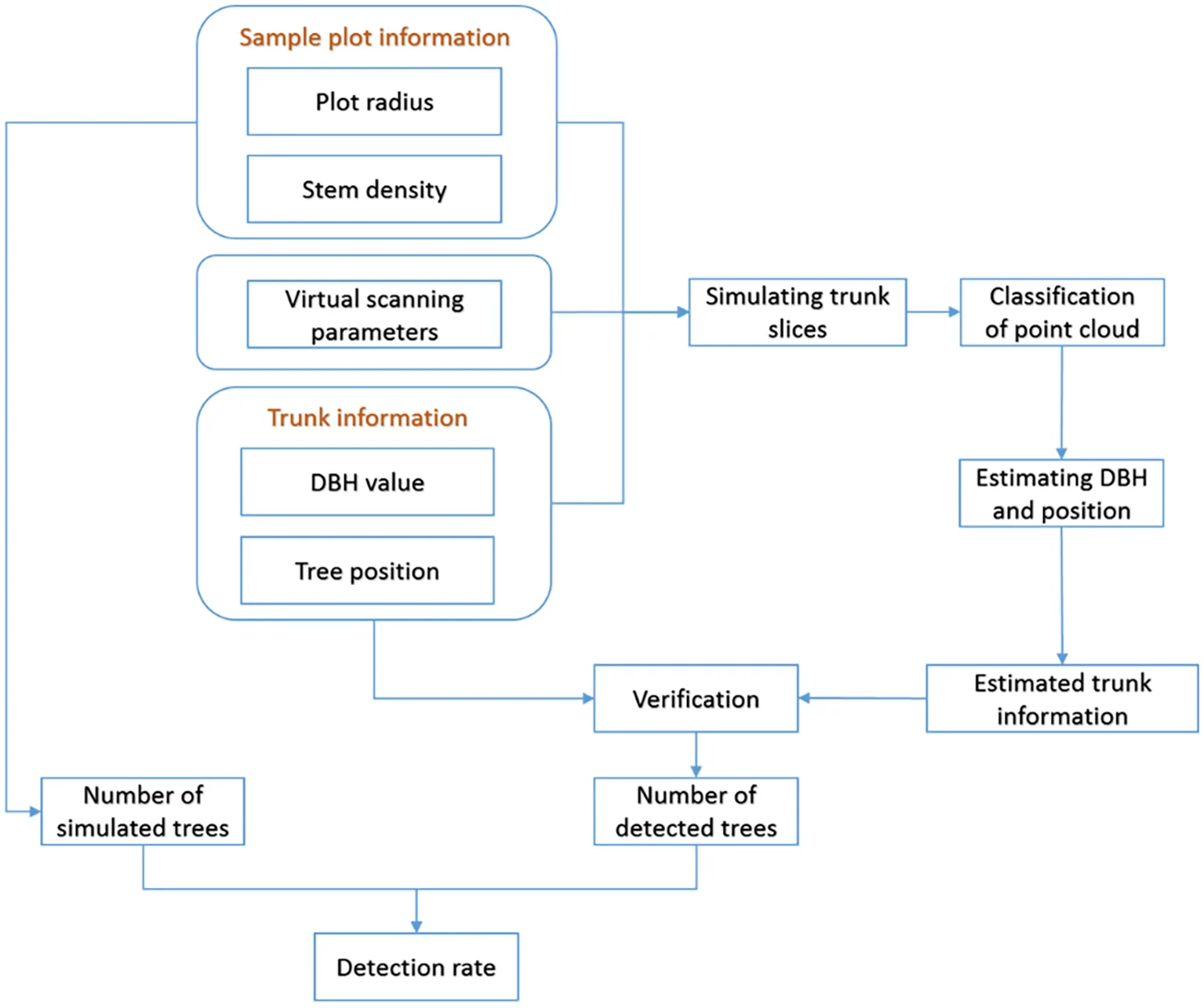
Flowchart of the point cloud simulation method for sample plot tree cross-sections. (Burt et al., 2019; Deng et al., 2024; Kuželka et al., 2020; Wan et al., 2019; Wang et al., 2019; Yrttimaa et al., 2020).

To facilitate a systematic analysis of tree detection rates, we constructed a simplified simulation model, as illustrated in **Figure 4**. The model incorporates three independent sets of input parameters. Consistent with standard forest inventory practices and the field survey design adopted in this study, all simulations were conducted using circular sample plots. The first parameter set defines plot-level characteristics, including plot radius and stem density. The second set describes tree structural attributes, specifically DBH and spatial positions. The third set specifies virtual LiDAR scanning parameters.

We introduced simplifying assumptions to construct a tractable simulation model: The virtual TLS scanner is positioned 1.3 m above ground level, the standard height for DBH measurements. A 3 m radius tree-free buffer zone is established around the scanner to eliminate near-field scanning artifacts. All virtual scanning parameters are held constant across simulations to isolate the effects of forest structural variables. Tree DBH and center position are estimated from a single horizontal height slice at 1.3 m above ground level.

**Figure 5:**
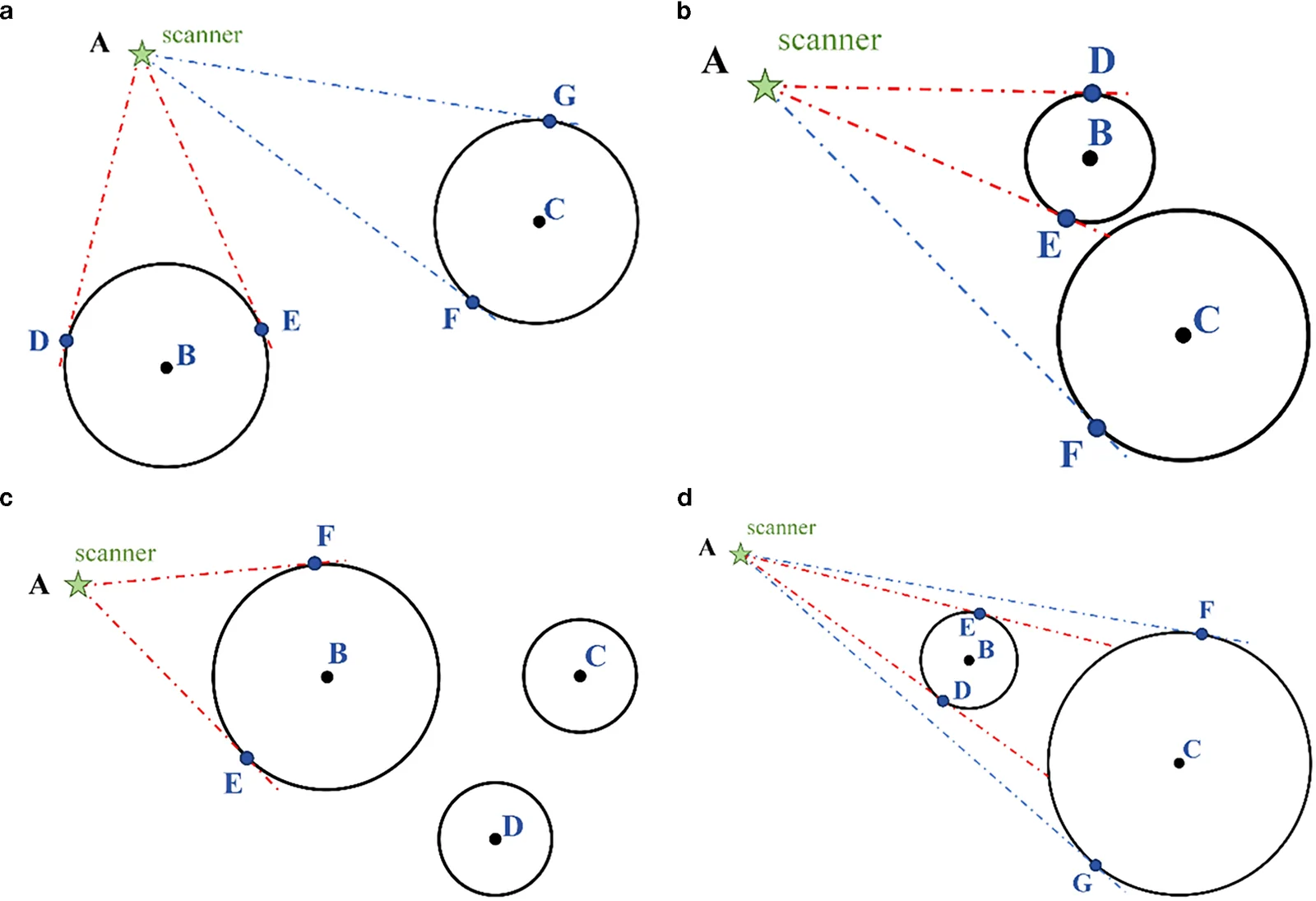
Flowchart of the point cloud simulation method for sample plot tree cross-sections. **(a)** Trees B and C do not obscure each other; **(b)** Tree B partially obscures one side of Tree C; **(c)** Tree B completely obscures Tree C and Tree G; **(d)** Tree B obscures the middle portion of Tree C.

Under these assumptions, point cloud slices were generated based on the scanning geometry and relative tree positions. Inter-tree occlusion resulted in varying degrees of scan completeness, with trees falling into four distinct categories. (**Figure 5**): (A) fully visible trees with no occlusion; (B) partially occluded trees with continuous point clouds; (C) completely occluded trees with no detectable points; and (D) fragmented trees split into multiple disconnected point cloud segments.

The first three categories were also demonstrated in the study of Kuronen et al. (2019). However, the fourth occlusion category refers to the incomplete shielding of distant large trees by nearby small trees. When the exposed left and right segments of the occluded large trees can both be detected in TLS scanning, the dection results need to be merged after tree detection.

The simulated point cloud slices were first segmented into clusters using density-based clustering. Each cluster was interpreted as a potential tree trunk slice, and circle fitting was applied to estimate the corresponding DBH and trunk center coordinates (Cabo et al., 2018). The resulting tree estimates were then matched against the ground truth tree inventory. A successful detection was declared if an estimate fell within a predefined distance threshold of a ground truth tree. Conversely, estimates with no corresponding ground truth were classified as false positives, and ground truth trees with no matching estimates were classified as false negatives.

After processing all point cloud clusters using the above detection pipeline, the number of detected trees per plot, *N_d_*, was determined. The DR for each sample plot was then calculated as follows:

(8) $$DR=N_{d}/N_{sim}$$

Using the full ensemble of virtual sample plots, we systematically varied the model input parameters to statistically evaluate their individual and combined impacts on detection performance.

### 3.4 Tree detection

In our simulations, we neglected range and angular measurement errors to ensure perfect point positioning accuracy. Based on the LiDAR scanning geometry, points within the same trunk slice exhibit characteristic spacing patterns determined by the scanner’s vertical and horizontal angular resolutions and the distance from the scanner to the trunk. These inherent spacing properties were used to effectively segment point clouds into individual trunk slices.

**Figure 6:**
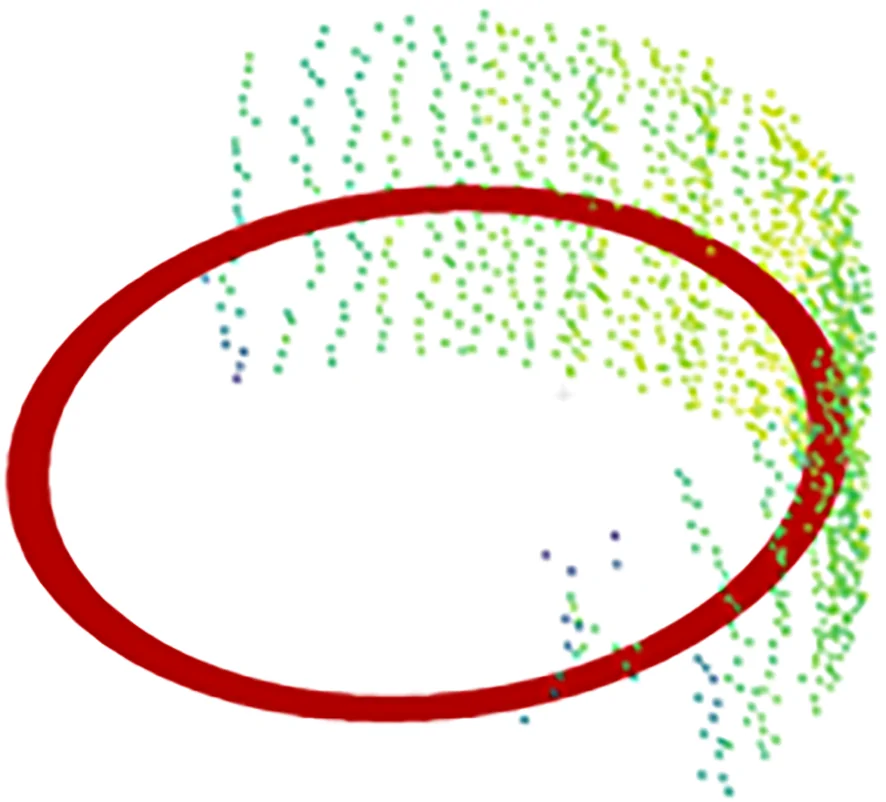
3D fitting schematic diagram of point cloud. The green part is the cylindrical point cloud image of tree trunks, and the red circles represent the tree DBH fitted based on the scanning results.

Circle fitting was applied to each segmented trunk slice to estimate tree DBH and trunk center coordinates. Theoretically, a circle can be uniquely defined by three non-collinear points in the absence of measurement errors. In this study, we employed the Taubin circle fitting algorithm, which offers an optimal balance of computational efficiency and fitting accuracy for TLS data (Cabo et al., 2018). The diameter and center of the fitted circle were taken as the estimated DBH and trunk position, respectively (**Figure 6**).

Detection performance was evaluated by comparing estimated trunk parameters against will be with the ground truth trunk inventory. A tree was considered successfully detected if its estimated parameters matched a corresponding ground truth entry within the predefined threshold. The overall tree detection rate was then calculated as the ratio of the number of detected trees to the total number of ground truth trees in the plot.

## 4 Results

### 4.1 Simulated data with fixed and random DBH values

**Figure 7:**
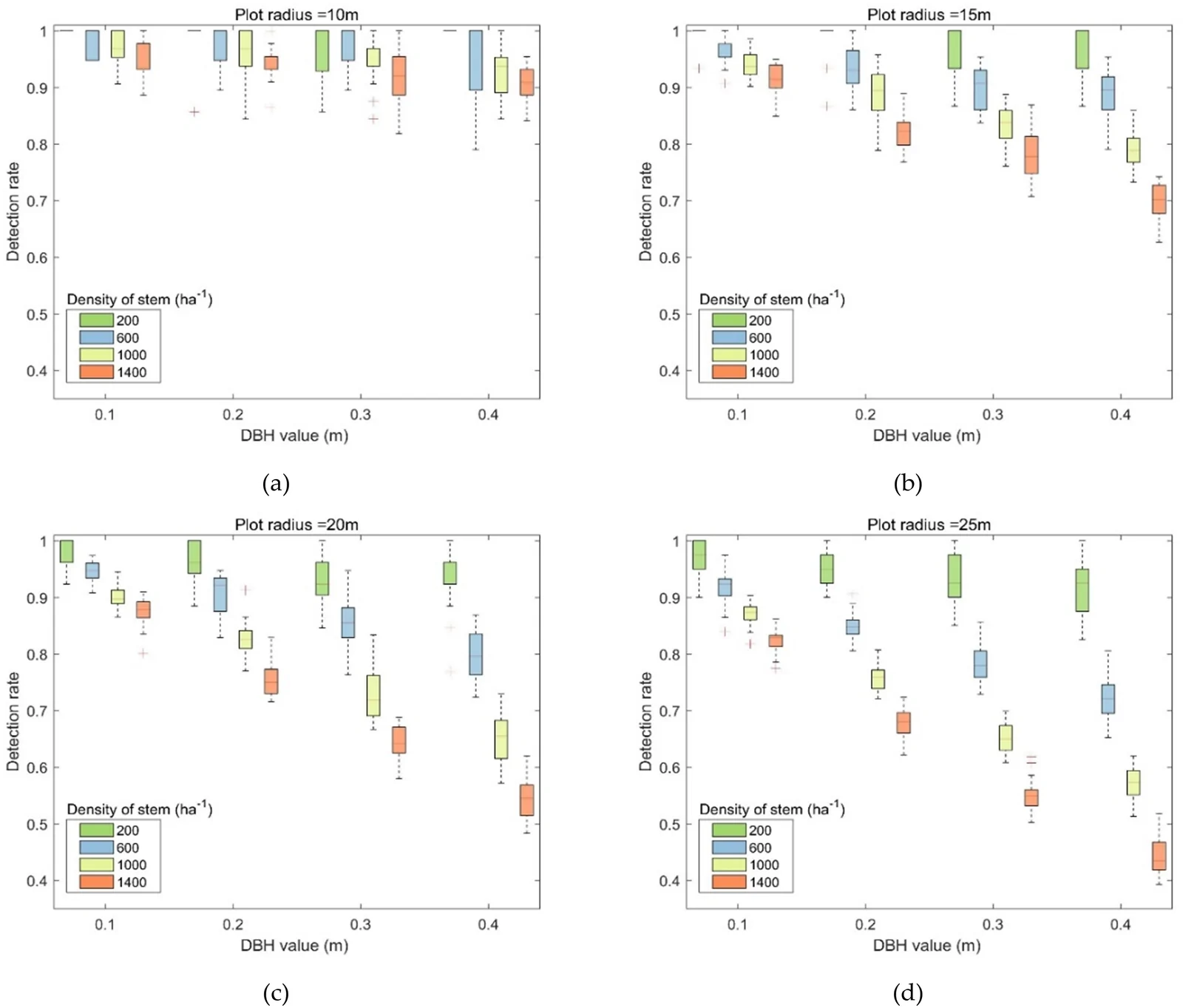
The impact of changes in DBH and stem density on detection rate when the sample plot size is fixed. **(a)** Plot radius=10m; **(b)** Plot radius=15m; **(c)** Plot radius=20m; **(d)** Plot radius=25m.

**Figure 8:**
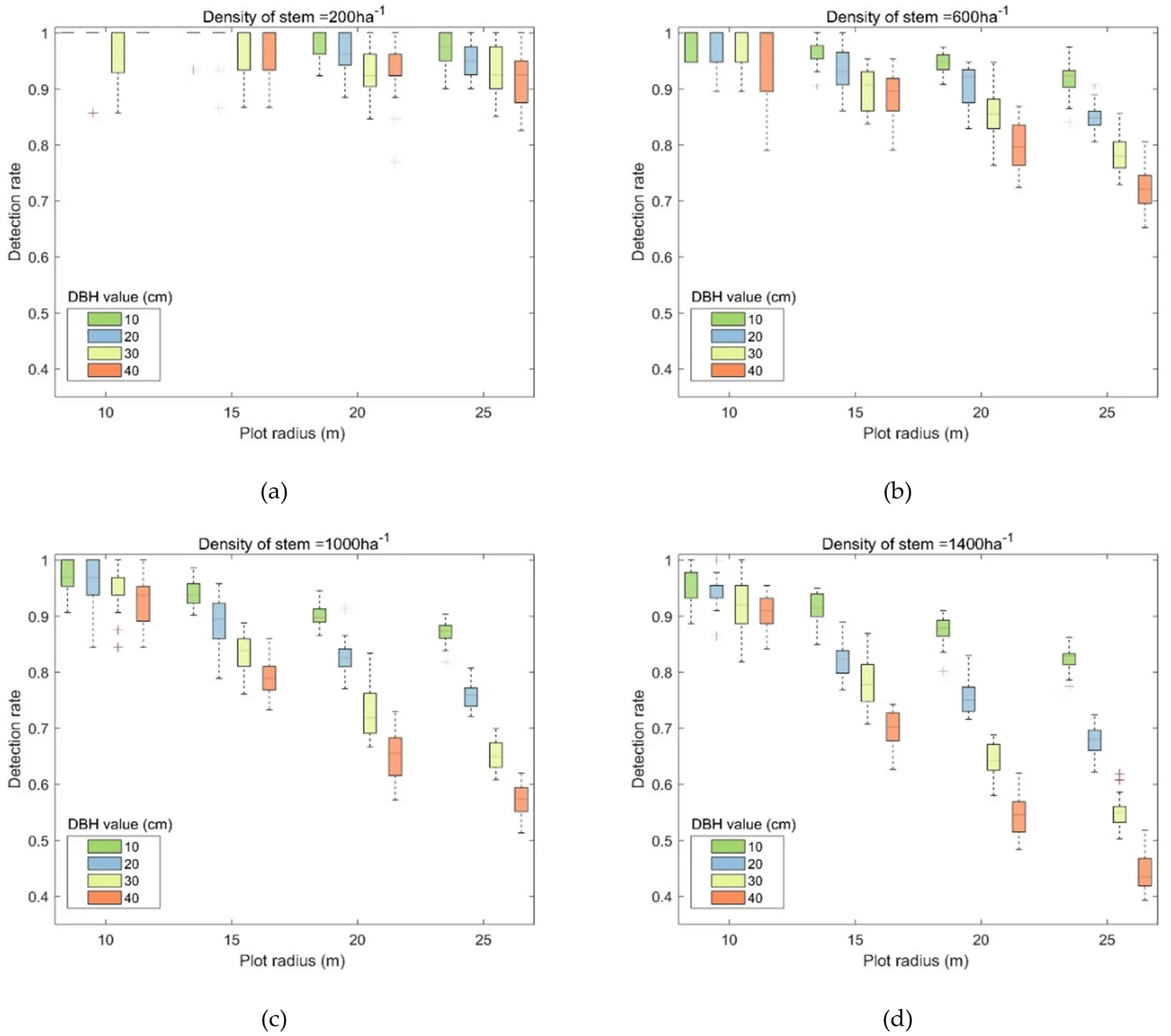
The impact of changes in DBH and sample plot size on detection rate when stem density is fixed. **(a)** Density of stem=200ha-1; **(b)** Density of stem=600ha-1; **(c)** Density of stem=1000ha-1; **(d)** Density of stem=1400ha-1.

**Figure 9:**
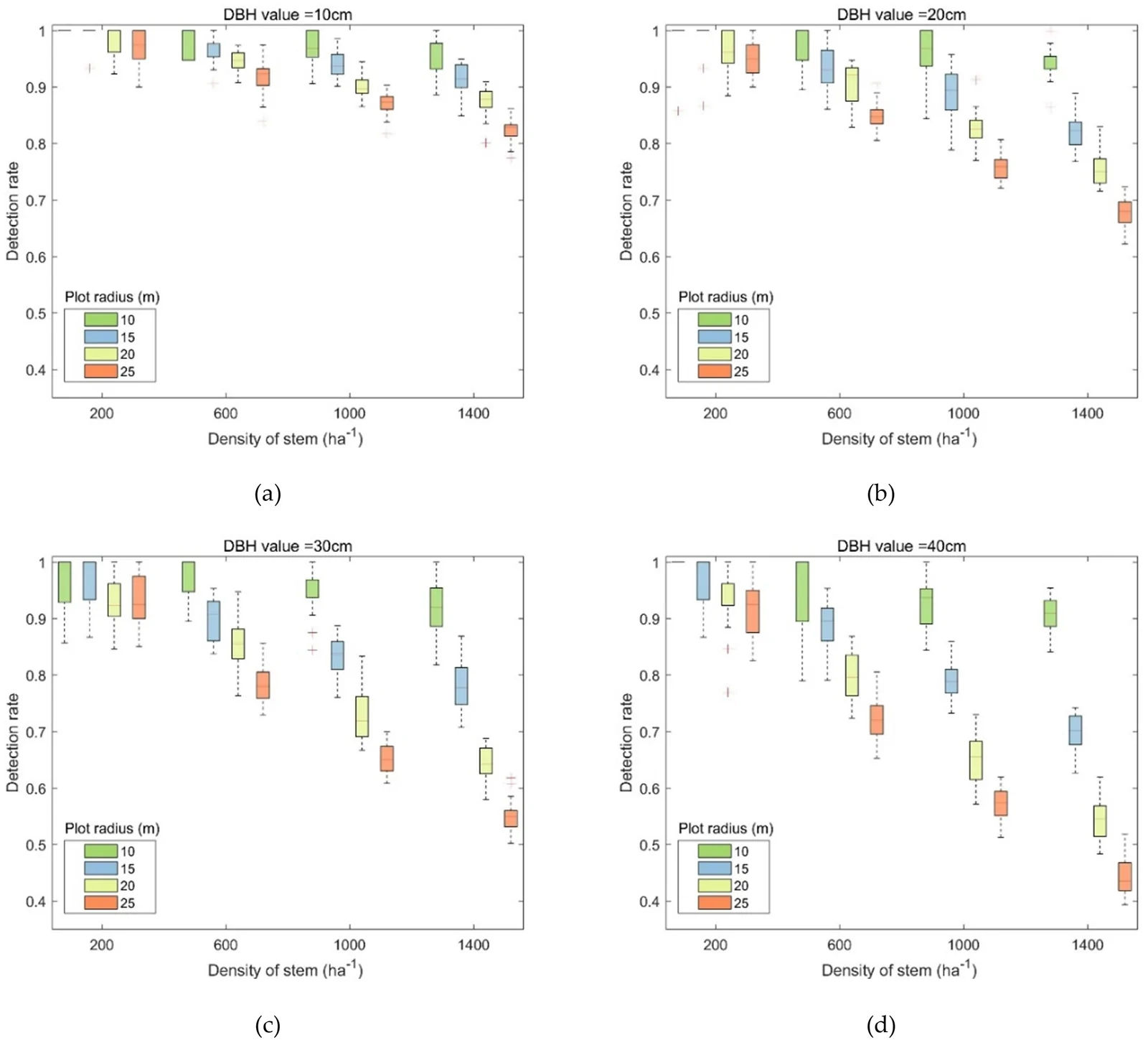
The impact of changes in stem density and sample plot size on detection rate when DBH is fixed. **(a)** DBH value =10cm; **(b)** DBH value =20cm; **(c)** DBH value =30cm; **(d)** DBH value =40cm.

We first analyzed detection rates across the full factorial design of fixed DBH simulations, comprising 1280 independent virtual plots (64 parameter combinations × 20 replicates each). Results are visualized as box-and-whisker plots in **Figures 7**–**9**. Figure of simulation data results.

The results revealed a clear and consistent trend: tree detection performance improved significantly when any of the three key structural parameters was minimized. At a stem density of 200 stems ha^-^¹, the lowest level tested, all replicates achieved detection rates above 80% regardless of plot radius and DBH (**Figure 8a**). Similarly, median detection rates exceeded 80% for plots with 10 m radius (**Figure 7a**) or 0.1 m DBH (**Figure 9a**).

In contrast, detection rates declined sharply with increasing plot radius, stem density, and DBH. This decline was nonlinear, with the steepest drops observed at the highest parameter values. Under the most extreme conditions tested (25 m plot radius, 1400 stems ha^-^¹ stem density, and 0.4 m DBH), the median detection rate fell to about 40%. This pattern is directly consistent with our theoretical occlusion model: larger DBHs increase the angular extent of each tree, higher stem densities increase the number of potential occluders, and trees farther from the scanner have smaller angular separations, making them more susceptible to mutual occlusion.

**Figure 10:**
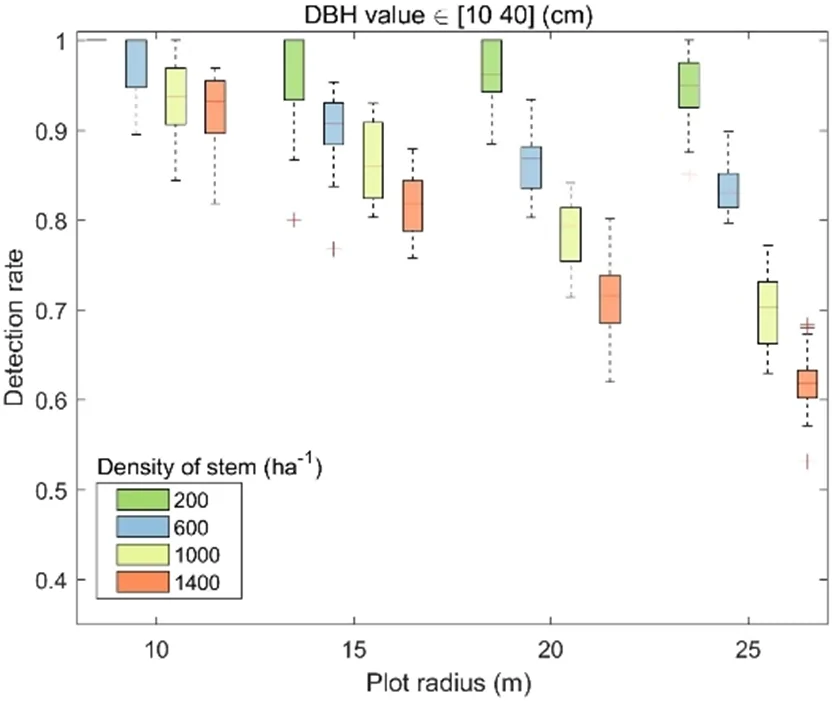
The impact of changes in stem density and sample plot size on detection rate when DBH is randomly selected within the range of 0.1 m to 0.4 m.

For simulations with DBH values randomly drawn from a uniform distribution (0.1–0.4 m), we observed the same qualitative dependence on plot radius and stem density (**Figure 10**). However, the random DBH dataset consistently outperformed the 0.4 m fixed DBH scenario across all plot sizes and stem densities. Even at the highest stem density of 1400 stems ha^-^¹, the median detection rate remained above 52%, compared to 40% for the fixed 0.4 m DBH case. This difference arises because the random DBH distribution includes a significant fraction of smaller trees, which are less likely to occlude or be occluded by neighboring trees.

### 4.2 Comparison between theoretical calculation and simulation results

**Figure 11:**
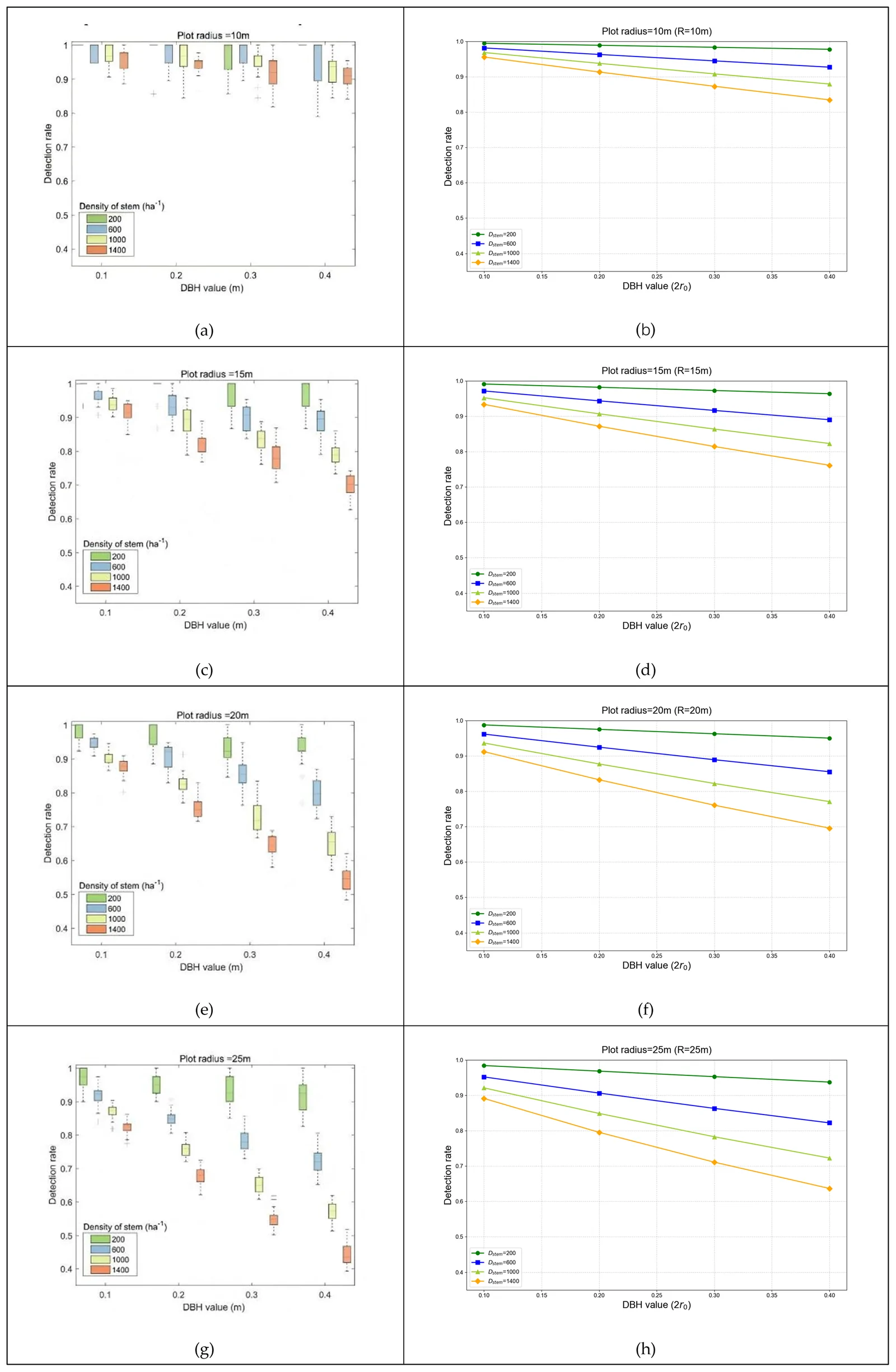
Results comparison between theoretical calculation and experimental results. **(a, b)** the detection rates from point cloud simulation and mathematical model when Plot radius=10m, respectively; **(c, d)** the detection rates from point cloud simulation and mathematical model when Plot radius=15m, respectively; **(e, f)** the detection rates from point cloud simulation and mathematical model when Plot radius=20m, respectively; **(g, h)** the detection rates from point cloud simulation and mathematical model when Plot radius=25m, respectively.

To validate the consistency between our theoretical model and simulation experiments, we compared the simulated detection rates with the predictions derived from Equation 8 (**Figure 11**). Both datasets showed consistent decreasing trends in detection rate with increasing DBH, plot radius, and stem density. The theoretical model predictions closely matched the simulation results, particularly for small plot radii.

However, theoretical DR was consistently higher than those obtained from point cloud simulations. This discrepancy increased monotonically with larger plot radii and higher stem densities. In most cases, the absolute difference between theoretical and simulated detection rates was less than 10%. The two datasets converged almost perfectly at small plot radii and low stem densities, but diverged to approximately 20% under the most challenging conditions (25 m plot radius, 0.4 m DBH, and 1400 stems ha^-^¹ stem density).

We attribute this systematic discrepancy to a key simplifying assumption in our mathematical model: that any tree not completely occluded will be detected. In reality, successful detection requires that the unobscured portion of the trunk contains sufficient points to form a reliable circle fit. Additionally, while our simulations neglected measurement noise, real LiDAR systems introduce small positional errors. Both factors become increasingly significant at larger distances and higher stem densities, leading to the observed divergence between theoretical predictions and simulation results.

### 4.3 Field test results

We conducted tree detection rate tests on five field plots under four different plot radius settings, with detailed results presented in **Tables 4**, **5**. At 10 m radius, detection rates ranged from 0.82 to 1.00, with three plots achieving rates ≥0.96 and an overall average of 0.95—the highest among all radius gradients. At 15 m radius, detection rates clustered between 0.90 and 0.94 with minimal variation, yielding an average of 0.91 (a 0.04 decrease from 10 m). At 20 m radius, detection rates dropped to 0.82–0.88, with an average of 0.85 (a further 0.06 decrease). At 25 m radius, detection rates varied from 0.77 to 0.86 with increased variability, and the average fell to 0.80—the lowest value observed.

**Table 5:** The information of the detection rate field test.

Station	Radius	Real tree number	Estimating tree number	Miss tree number	Detection rate
1	10	27	27	0	1
2	10	28	26	3	0.82
3	10	24	23	0	0.96
4	10	27	26	0	0.96
5	10	21	21	0	1
Average	10				0.95
1	15	61	55	0	0.9
2	15	64	63	3	0.94
3	15	59	53	0	0.9
4	15	62	56	0	0.9
5	15	58	54	0	0.93
Average	15				0.91
1	20	107	89	1	0.82
2	20	112	101	6	0.85
3	20	106	96	3	0.88
4	20	113	97	0	0.86
5	20	114	92	0	0.84
Average	20				0.85
1	25	168	146	1	0.86
2	25	175	140	6	0.77
3	25	174	161	19	0.82
4	25	177	136	0	0.77
5	25	174	144	9	0.78
Average	25				0.8

As plot radius increased from 10 m to 25 m, the average tree detection rate decreased monotonically from 0.95 to 0.80. Concurrently, the variability in detection rates across individual plots increased, indicating lower stability for larger-radius surveys.

A three-way comparison of theoretical predictions, simulation results, and field measurements showed that both theoretical and simulated detection rates accurately reflected real-world performance. The theoretical formula provided the most accurate estimates under conditions of small plot radius, low stem density, and small DBH, with errors increasing at larger radii, higher densities, and larger DBHs. Discrepancies arose from two primary sources: (1) at small radii, the limited number of trees per plot leads to high statistical variability in occlusion effects; (2) at large radii, increased presence of shrubs, leaves, and other vegetation in point cloud data introduces more false detections.

Overall, our theoretically derived detection rate formula provides reliable estimates of tree detection performance for TLS field surveys.

## 5 Discussion

Our simulation results demonstrate that plot radius, stem density, and DBH all negatively affect tree detection rates in ground-based LiDAR surveys. This finding is consistent with the conclusions reported in the study by Olofsson and Olsson (2018). Specifically, larger plot radii increase the average distance between trees and the scanner, thereby leading to higher occlusion probabilities. When stem density is constant, trees added by expanding plot size are disproportionately located farther from the scanner and have more potential occluders in their line of sight, further exacerbating detection failure. Higher stem densities increase the frequency of inter-tree occlusion, as laser pulses are more likely to be intercepted by foreground trees before reaching background individuals. Larger DBHs increase the cross-sectional area of trees, enhancing their ability to block laser beams. While larger trees themselves are generally easier to detect, they act as stronger occluders for neighboring trees, reducing overall stand-level detection rates. Among the three factors, stem density exerts the strongest and most direct influence: detection rates remain consistently high at 200 stems ha^-^¹ but decline sharply with increasing density and DBH, except for the smallest 10 m radius plots.

Although reducing any of these three parameters improves detection performance, stem density and DBH are inherent properties of forest stands and cannot be adjusted during survey design. Plot radius is therefore the only controllable variable for optimizing detection accuracy. Our results indicate that smaller plot radii are preferable for ground-based LiDAR surveys: at 10 m radius, detection rates exceed 80% across nearly all stand conditions and are least sensitive to variations in stem density and DBH. This aligns with the conclusion from Olofsson and Olsson (2018), who also suggested in their study that a 10m plot size ensures favorable performance for the majority of TLS detection algorithms.

Importantly, our study demonstrates that for forests with uniformly and randomly distributed stem positions, the theoretical tree detection rate can be directly computed using Equation 7 given three key parameters: plot radius, stem diameter, and total stem count within the sample plot. Despite potential deviations between actual and theoretical detection rates, this theoretical value can serve as a quantitative reference for researchers to plan the experiment or verify whether experimental results align with theoretical expectations.

Both our point cloud simulations and theoretical model are based on simplifying assumptions that neglect terrain effects, non-random tree distributions, understory vegetation interference, and algorithm-specific limitations. These factors generally reduce detection rates in real-world conditions, meaning that actual field detection rates are typically lower than our simulated values.

To overcome the limitation of discrete parameter combinations in simulation experiments, we developed an analytical mathematical model based on the geometric relationships. The model predictions show excellent overall agreement with simulation results, with discrepancies exceeding 10% only under the most challenging conditions (large plot radii, high stem densities, and large DBHs). Therefore, our model can be directly applied to estimate theoretical detection rates for most field survey scenarios, providing a valuable benchmark for evaluating the quality of field-derived detection results.

Nevertheless, it should be noted that this study is currently limited to simulation scenarios with uniformly distributed stem positions, where stem diameters are either fixed at a constant value or randomly varied within a predefined range. Such simplified settings differ notably from real-world forest conditions, particularly natural forests with complex stand structures, meaning the proposed models and theoretical frameworks still require further improvement and refinement.

In particular, significant deviations between model estimates and field measurements may arise under more complex forest conditions, such as clustered tree distributions or rugged terrain. Given the strong extensibility of the proposed model framework, future work can extend the current system to incorporate inhomogeneous tree spatial distributions, heterogeneous DBH structures, and irregular plot geometries to further enhance the accuracy and generalizability of detection rate predictions. Moreover, the model can be further validated and optimized against international benchmark datasets covering complex forest structures and terrain conditions.

## 6 Conclusion

This study investigated the effects of plot radius, stem density, and DBH on TLS tree detection rates via point cloud simulations and theoretical modeling. All three factors negatively affect detection rates by increasing inter-tree occlusion, with stem density exerting the strongest influence (exceeding 80% at 200 stems ha^-^¹ and declining sharply at higher densities). Smaller plot radii are the only controllable parameter, which can significantly improve detection accuracy and reduce sensitivity to stand structural variation.

The derived geometric model shows good overall agreement with simulations and provides a practical tool for homogeneous forests, with discrepancies only under extreme conditions (large radii, large DBH, high density). We recommend prioritizing smaller plot radii for TLS forest inventories. The model can serve as a reliable baseline for field result evaluation and pre-survey planning. Future research will extend the framework to complex forest conditions to enhance real-world applicability. This study provides theoretical support for TLS-based tree detection and optimizes forest inventory plot design.

## Data Availability

The raw data supporting the conclusions of this article will be made available by the authors, without undue reservation.
